# New Perspective in Diabetic Neuropathy: From the Periphery to the Brain, a Call for Early Detection, and Precision Medicine

**DOI:** 10.3389/fendo.2019.00929

**Published:** 2020-01-17

**Authors:** Heng Yang, Gordon Sloan, Yingchun Ye, Shuo Wang, Bihan Duan, Solomon Tesfaye, Ling Gao

**Affiliations:** ^1^Endocrinology Department, Renmin Hospital of Wuhan University, Wuhan, China; ^2^Diabetes Research Unit, Sheffield Teaching Hospitals, Royal Hallamshire Hospital, Sheffield, United Kingdom

**Keywords:** diabetic neuropathy, painful diabetic neuropathy, stratified medicine, diagnosis diabetic neuropathy, painful diabetic neuropathy treatment

## Abstract

Diabetic peripheral neuropathy (DPN) is a common chronic complication of diabetes mellitus. It leads to distressing and expensive clinical sequelae such as foot ulceration, leg amputation, and neuropathic pain (painful-DPN). Unfortunately, DPN is often diagnosed late when irreversible nerve injury has occurred and its first presentation may be with a diabetic foot ulcer. Several novel diagnostic techniques are available which may supplement clinical assessment and aid the early detection of DPN. Moreover, treatments for DPN and painful-DPN are limited. Only tight glucose control in type 1 diabetes has robust evidence in reducing the risk of developing DPN. However, neither glucose control nor pathogenetic treatments are effective in painful-DPN and symptomatic treatments are often inadequate. It has recently been hypothesized that using various patient characteristics it may be possible to stratify individuals and assign them targeted therapies to produce better pain relief. We review the diagnostic techniques which may aid the early detection of DPN in the clinical and research environment, and recent advances in precision medicine techniques for the treatment of painful-DPN.

## Introduction

Neuropathic syndromes are common complications of diabetes mellitus. By far the most prevalent is chronic diabetic peripheral sensorimotor neuropathy (DPN), affecting up to 50% of people with diabetes (1, 2). DPN is associated with increased mortality and leads to morbidity, principally as a result of its two major clinical consequences, diabetic foot ulceration, and neuropathic pain (3–5). Diabetic foot ulceration occurs as a result of a complex interaction of risk factors and patient behaviors, but sensory loss secondary to DPN is most often the primary cause (6). Lower-limb complications of diabetes are expensive and a substantial burden for patients, potentially leading to devastating outcomes such as lower limb amputation and death (3, 4, 6). Furthermore, up to half of patients with DPN suffer with painful neuropathic symptoms (painful-DPN) (7). These painful symptoms are commonly severe and often lead to depression, anxiety and sleep disorders, and reduced quality of life (8, 9).

Unfortunately, our understanding of the pathophysiology of DPN remains incomplete. Consequently, we do not have any effective disease modifying pharmacotherapies with which to treat the condition. The mainstay of modern management is to control risk factors for DPN, and prevent and manage its complications (10). Similarly, although a number of differences have been discovered between painless- and painful-DPN, the specific mechanisms causing the condition are unknown (11, 12). Disease modifying treatments are not widely used for painful-DPN and the treatment remains largely symptomatic (11, 12). Unfortunately, the available treatments for neuropathic pain are often ineffective and poorly tolerated (13). It has recently been hypothesized that by using certain patient characteristics (e.g., clinical features, quantitative sensory testing [QST], genetics and cerebral imaging) it may be possible to stratify individuals and assign them targeted therapies to produce better pain outcomes (14).

The prevalence of diabetes, DPN and foot amputations continue to increase at an alarming rate. It is essential that the condition is diagnosed early and accurately so that measures may be implemented to reduce the risk of diabetic foot complications. We review the recent advances in the diagnosis of DPN, which may supplement clinical assessment and could aid the early detection of DPN in the clinical and research environments, and precision medicine techniques, which may be used to improve the treatment of painful-DPN in the future.

## The Classification and Definition of Diabetic Neuropathies

Diabetic neuropathies are heterogenous in their clinical presentation, risk factors and pathophysiology. The neuropathic syndromes may be classified according to the nerve type affected (sensory vs. motor vs. autonomic), site of nerve injury (focal vs. multi-focal vs. generalized), and disease time course (acute vs. chronic) (2, 10, 15). The neuropathic syndromes may broadly be divided into typical DPN and atypical diabetic neuropathies, the latter of which are outside the scope of this review (16). The American Diabetes Association has recently developed a simplified classification schema for diabetic neuropathies, reproduced in Table 1 (10). Typical DPN is by far the most prevalent form of neuropathy in diabetes and characteristically affects both sensory and motor nerves in a peripheral distribution (1). However, the relative impact on small and large sensory fibers, and motor fibers varies among individuals. The Toronto Diabetic Neuropathy Expert Group defined DPN as “a symmetrical, length dependent sensorimotor polyneuropathy attributable to metabolic and microvessel alterations as a result of chronic hyperglycemia exposure (DM) and cardiovascular risk covariates (16).”

**Table 1:** Classification for diabetic neuropathies.

**A. Diffuse neuropathy**
DSPN
■ Primarily small-fiber neuropathy
■ Primarily large-fiber neuropathy
■ Mixed small- and large-fiber neuropathy (most common)
Autonomic
■ Cardiovascular: Reduced HRV, Resting tachycardia, Orthostatic hypotension, Sudden death (malignant arrhythmia) ■ Gastrointestinal: Diabetic gastroparesis; Diabetic enteropathy (diarrhea); Colonic hypomotility (constipation) ■ Urogenital: Diabetic cystopathy (neurogenic bladder), Erectile dysfunction, Female sexual dysfunction
Sudomotor dysfunction
■ Distal hypohydrosis/anhydrosis
■ Gustatory sweating
Hypoglycemia unawareness
Abnormal pupillary function
**B. Mononeuropathy (mononeuritis multiplex) (atypical forms)**
Isolated cranial or peripheral nerve (e.g., CN III, ulnar, median, femoral, peroneal) Mononeuritis multiplex (if confluent may resemble polyneuropathy)
**C. Radiculopathy or polyradiculopathy (atypical forms)**
Radiculoplexus neuropathy (lumbosacral polyradiculopathy, proximal motor amyotrophy) Thoracic radiculopathy

## Pathological Changes of Diabetic Neuropathy and Mechanisms

DPN leads to degenerative and atrophic changes throughout the peripheral and central nervous system (7, 17). The peripheral end terminals of nociceptors, intra-epidermal nerve fibers, are depleted in a distal symmetrical manner in DPN (7, 18). More proximally, peripheral nerve changes have been well-described and include; demyelination of myelinated nerve fibers, axonal degeneration and necrosis, Schwannopathy, and microangiopathy (19). Furthermore, autopsy and more recent advanced imaging studies have found spinal cord and cerebral atrophy associated with DPN (20–23).

**Figure 1:**
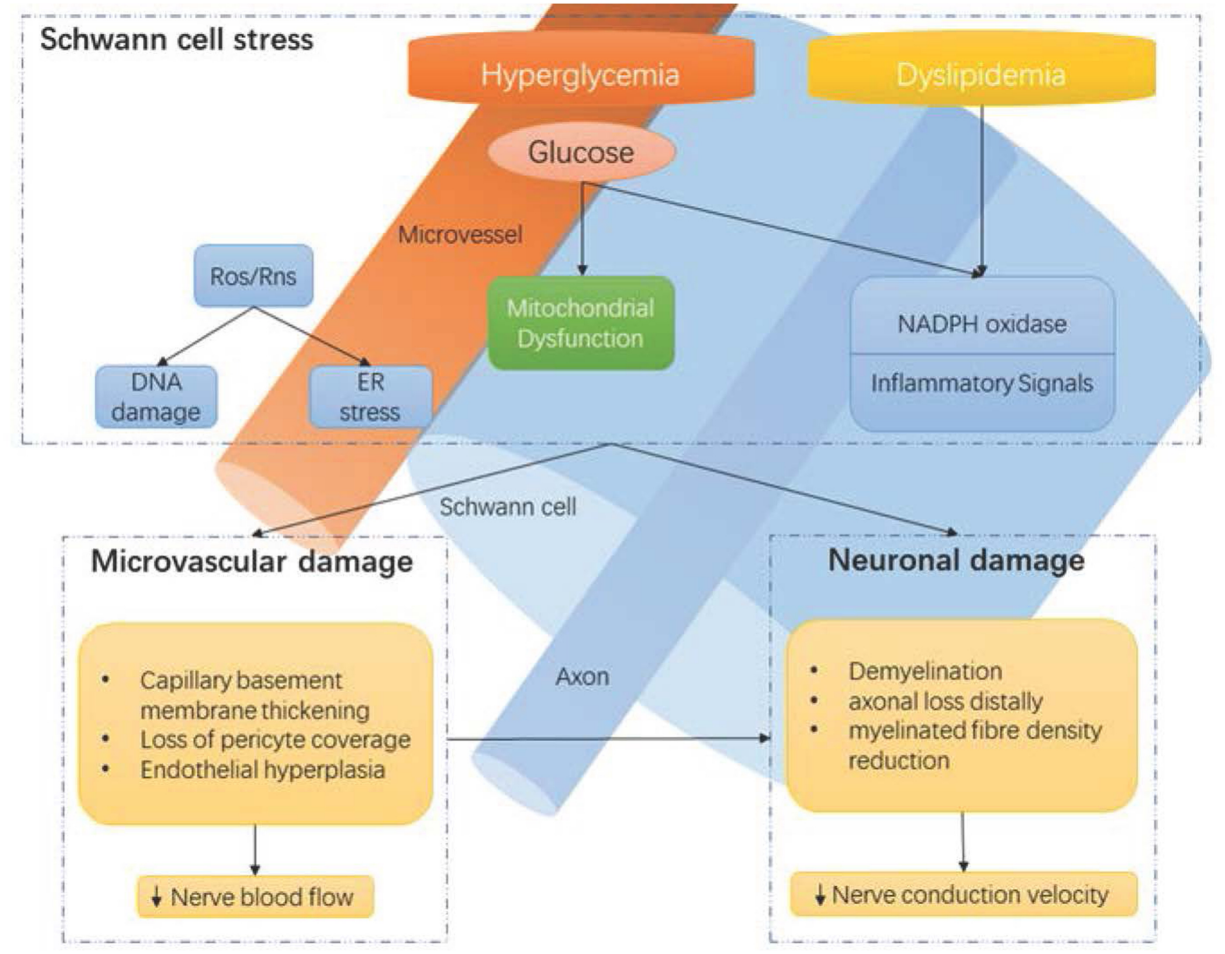
Hyperglycaemia-driven Schwann cell stress and neuronal damage. Hyperglycaemia and dyslipidemia lead to reduction of neuronal support from Schwann cells and microvessels. Disruption of neuronal support by Schwann cells and the vascular system contributes to neuropathy, in conjunction with the direct effects of hyperglycaemia on neurons. ER, endoplasmic reticulum; NADPH, Nicotinamide adenine dinucleotide phosphate; Ros, reactive oxygen species; Rns, reactive nitrogen species. Reproduced and permission gained from Sloan et al. (7).

A precise understanding of the pathophysiology of DPN remains elusive (24). A number of molecular pathways correlate with functional nerve impairment and pathological neuronal changes (Figure 1), including, but not limited to: polyol pathway activation, oxidative stress, protein kinase C activation, and advanced glycation end product formation (24, 25). However, the exact causal links between hyperglycemia and clinical DPN is uncertain. Our current understanding is that hyperglycemia, as well as vascular risk factors, activate detrimental pathways ultimately leading to downstream injury to the microvessel endothelium, nerve support cells, and nerve axons (25). Recent advances suggest that the cumulative effect of these injurious events may lead to neuronal death via reactive oxygen species generation and mitochondrial dysfunction. Furthermore, mechanistic and pathological findings do not discriminate between painful- and painless-DPN (12).

## Epidemiology of DPN

The prevalence of DPN, with or without pain, varies from study to study and is heavily dependent on the population selected, type of diabetes and case definition criteria used (26). Dyck et al. found that two thirds of patients with diabetes had objective evidence of some form of neuropathy (1). The most common was DPN, affecting ~50%. The duration of diabetes and glycemic control are the most significant risk factors for DPN (27). Other risk factors for cardiovascular disease are also associated with DPN, including: obesity, hypertension, smoking, and dyslipidemia (27–29).

Approximately 50% of people with DPN suffer with peripheral neuropathic pain (5, 29). Many risk factors for painful-DPN have been postulated such as the severity of neuropathy, hyperglycemic burden, and obesity (12). However, recent studies have demonstrated strong evidence that female sex is a risk factor for painful-DPN (12, 30). Idiopathic neuropathy is more prevalent in pre-diabetic states such as impaired glucose tolerance (IGT) (31). This lends further weight to the importance of vascular risk factors, such as the features of the metabolic syndrome other than hyperglycemia, playing an important role in the pathogenesis of peripheral neuropathy.

## Clinical Features of Diabetic Neuropathy

DPN may present with a wide range of clinical symptoms and signs. Some people may be entirely asymptomatic, where a foot ulcer can be the first presentation. However, other patients may experience one or a number of different symptoms such as paresthesia (tingling/pins and needles), numbness and neuropathic pain (often described as burning, lancinating, shooting, or aching) which can range from mildly troublesome to intractable, causing great suffering (32). These symptoms may be sporadic or constant, and their natural history varies among patients. Sensory symptoms may be present for only a short period of time before they disappear entirely, or they may become chronic. Sensory symptoms and clinical examination signs begin in the toes/distal foot symmetrically. On physical examination, light touch and pin-prick of the distal foot is commonly impaired first, followed by more advanced sensory (i.e., vibration and proprioception loss) and motor (i.e., weakness, clawing of the toes, ankle reflex loss, and loss of muscle bulk) abnormalities. As the disease progresses, it spreads proximally up the leg before impacting the finger tips and upper limbs. The physical examination for patients with painful-DPN is generally indistinct from those without neuropathic pain. However, some patients may have a pure small fiber neuropathy which results in a loss of small fiber modalities (i.e., pin-prick and temperature sensory loss) with normal large fiber function (16). Additionally, a small sub-set of patients have the so called “irritable nociceptor” phenotype with “positive” sensory signs such as allodynia and hyperalgesia (33, 34).

## Diagnosis of DPN

The diagnosis of DPN is often made during diabetic foot screening. Type 2 diabetes is often diagnosed after it has been present for some time; therefore, patients with type 2 diabetes should be screened for DPN from diagnosis (10). However, the risk of DPN is low at diagnosis of type 1 diabetes, so foot screening should commence 5 years after diagnosis. Subsequently, all patients should be assessed on an annual basis for lower limb sensory and vascular deficits (10). Once there is a clinical suspicion of DPN, a thorough clinical assessment must exclude other causes of neuropathy, and should involve a comprehensive history and examination including: temperature/pinprick sensation testing to assess small-fiber function; vibration sensation testing with 128-Hz tuning fork and assessment of ankle reflexes to assess large fiber function; and 10-g monofilament for the assessment of protective sensation. Clinical scoring systems may also be used to aid in diagnosing DPN e.g., Toronto Clinical Scoring System (35). Routine biochemical assay should be performed to determine the quality of glycemic and cardiovascular risk factor control and rule out other causes of peripheral neuropathy (e.g., coeliac disease, vitamin B12 deficiency, hypothyroidism, infectious/inflammatory disease). When the clinical features are atypical or the diagnosis is unclear then patients should be referred for specialist assessment. Nerve conduction studies remain the gold standard measure of large fiber function, but QST and skin biopsy may be used for diagnosing small fiber neuropathy (16).

Unfortunately, by the time clinical DPN is diagnosed irreversible nerve injury has already taken place. More advanced diagnostic techniques may be able to diagnose the condition at an early stage. Additionally, these methods may play an important role in clinical research as they may be more sensitive to changes in nerve function than current clinical measures and could be used as endpoints to assess the efficacy of pathogenetic treatments in clinical trials.

## Skin Biopsy and Quantification of Intra-epidermal Nerve Fiber Density

Skin biopsy of the distal leg with quantification of intra-epidermal nerve fiber density (IENFD) is the gold standard technique to diagnose small fiber neuropathy (SFN) and it is also recommended for diagnosing DPN (16, 36). The procedure involves infiltration of subcutaneous local anesthetic and removal of a small skin sample using a punch biopsy tool. The sample must be immediately fixed, prepared and then epidermal innervation is quantified using either immunofluorescent or immunohistochemistry microscopy. The biopsy itself is quick and easy to perform but it is necessary to have suitable laboratory equipment and expertise to analyse. The technique is minimally invasive with a low complication rate, infection occurs in ~1 in 1,000.

IENFD correlates with other measures of neuronal function and has a sensitivity of 61–90% and specificity 64–82.8% for diagnosing DPN (37–43). The natural rate of epidermal innervation depletion is accelerated in DPN and IENFD may act as an early marker for DPN (44, 45). Despite being a measure of nociceptor density in the epidermis, IENFD is not related to the presence or intensity of neuropathic pain (7, 12). However, recent studies indicate that IENF regeneration and dermal vasculature differentiate painless- from painful-DPN (7, 45–47). Due to its invasive nature, skin biopsy with IENFD quantification is unlikely to be an appropriate screening tool for DPN. However, it has utility in clinical and research environments as a diagnostic tool. Moreover, IENFD has also been used as a clinical endpoint, Smith et al. found that diet and exercise counseling of patients with pre-diabetic neuropathy could lead to improvements in IENFD which corresponded with improvement in neuropathic pain (48). Further validation is required before IENFD can be used as a suitable biomarker for clinical trials in DPN (49).

## Corneal and Retinal Innervation

A number of different ophthalmic measures of neuronal integrity have been proposed as surrogate measures of DPN and other neurological diseases, including corneal confocal microscopy (CCM), retinal nerve fiber layer thickness, and pupil responsiveness (50–52). CCM is a rapid and non-invasive modality for the study of corneal innervation and has emerged as a technique for diagnosing DPN (53). It has a high sensitivity (68–92%) and a specificity of 40–64% to diagnose DPN (54–56). Furthermore, CCM measures correlate with IENFD on skin biopsy (38). Pritchard et al. demonstrated that a reduced corneal nerve fiber length was predictive of incident DPN (57). Moreover, Dehghani et al. found that corneal nerve parameters rapidly declined prior to the development of foot complications (58).

Optical coherence tomography (OCT) has been used to identify the loss of retinal nerve fibers in a number of neurological disease, including DPN (50). Retinal nerve fiber layer (RNFL) loss is observed in patients with diabetes and correlates with the stage of diabetic retinopathy (59, 60). However, reports have shown that RNFL loss in patients with diabetes without diabetic retinopathy (61, 62). Indeed, two recent studies have found that measures of RNFL loss are associated with DPN (60, 63). OCT and CCM measures hold promise as a reliable and repeatable non-invasive measure which may be used to detect early DPN in the clinical and research setting. However, they are not currently widely available as they require specialist expertise and expensive equipment to perform (50).

## Neurometer

The Neurometer is a non-invasive neurodiagnostic, QST device to measure current perception threshold (CPT) (64). It selectively determines the functional status of three nerve types, large myelinated (Aβ) fibers, medium-size myelinated (Aδ) fibers, and unmyelinated (C) fibers by measuring CPT at 2,000, 250, and 5 Hz, respectively (65). The device is quick, painless and can detect hypo- and hyper-aesthesia (64). Studies have found that measurement of the CPT using the Neurometer detects milder DPN more sensitively than vibration perception threshold (VPT) (66, 67) and Monofilament testing (66, 68). A recent study enrolled 202 patients with type 2 diabetes mellitus and compared clinical phenotyping using the CPT against a clinical scoring system (Michigan Neuropathy Screening Instrument; MNSI) and nerve conduction studies (NCS) (69). NCS variables differed across CPT clinical phenotypes. However, the study found that NCS detected more cases of subclinical DPN than the Neurometer. Furthermore, Matsutomo et al. found that the neurometer identified dysfunction of myelinated, but not unmyelinated fibers, in the diagnosis of DPN (65). Additionally, Koo et al. found that although the CPT correlated with neuropathic symptoms and signs it provides little additional information compared with conventional testing (70). As with other QST techniques, CPT abnormalities are not specific to DPN, and the test may be influenced by other psychological factors.

## DPN-Check

DPN-Check is a handheld point-of-care device which provides the sural nerve amplitude and conduction velocity without the need for neuroelectrophysiologist expertise or expensive equipment. It is user-friendly and requires only basic training to use. The device stimulates the sural nerve orthodromically with distal probes, as opposed to antidromically as in standard NCS protocols, and records using a biosensor covering a wide area of the lower limb proximally. It has a sensitivity of 95 and 71% specificity to diagnose DPN (71). Additionally, it demonstrates inter-rater and intra-rater reliability and performs well in comparison to clinical examination and laser-doppler “FLARE” imaging (71, 72). The DPN-Check sural nerve amplitude measurements demonstrate strong agreement with standard NCS; however, DPN-Check over-estimates sural nerve conduction velocity (71). Additionally, any sural nerve amplitudes below 1.5 μV are adjusted to zero. Although further work to determine the generalizability to the clinical and research setting is required, this simple technique has potential to accurately measure sensory nerve function quickly and cheaply (73). A recent study by Binns-Hall et al. demonstrated that the DPN-check was effectively used to detect early DPN during combined eye, foot and retinal screening visits (74).

## Sudomotor Testing

The foot sweat glands are innervated by sudomotor, unmyelinated cholinergic nerve fibers which may become impaired in DPN (16). Sudomotor dysfunction leads to foot skin dryness which is associated with an elevated risk of foot ulceration (75). There are several methods to determine sudomotor function in DPN, including: quantitative sudomotor axon reflex test, thermoregulatory sweat test and the quantitative direct and indirect reflex test (16). Neuropad is a sudomotor functional index test. It is a simple patch applied to the skin whose color changes from blue to pink through chemical reaction to evaluate sudomotor function (76). The presence of neuropathy is determined by color change after the patch has been adhered to the skin for 10 min. It has a sensitivity ranging from 86 to 95% but a specificity of only 45–69.8% for diagnosing DPN (77–80). A more recent study found that automated quantification of Neuropad improves the diagnostic ability of the test, especially for peripheral small fiber neuropathy (81). Neuropad is easy to use and provides a non-subjective result but its relatively poor specificity limits its applicability.

A more recent sudomotor testing device is Sudoscan, which is a non-invasive, FDA-approved device for the diagnosis of DPN. It measures the electrochemical skin conductance (ESC) of the hands and feet by reverse iontophoresis to objectively measure sudomotor function (82). It is quick and easy to perform and has a sensitivity ranging between 70 and 87.5%, and specificity 76.2–92%, to detect DPN (82–84). A recent large cross sectional study found ESC as measured by Sudoscan to be the most sensitive measure (Area under receiver-operator characteristic curve plot 0.88) for the early detection of DPN in comparison to VPT and clinical assessment (85). Additionally, it has a similar diagnostic utility as skin biopsy with IENFD measurement and it correlates with other measures such as clinical neuropathy scoring systems, QST, autonomic function testing and NCS parameters (82–86). However, a recent systematic review concluded that there was insufficient evidence to support that Sudoscan as a measure of sensory nerve fiber function, listing conflicts of interests, inconsistent normative values and insufficient sensitivity and specificity from pooled data-sets (87). Further validation is required to determine the value of sudomotor testing in predicting clinically relevant outcomes such as foot ulceration to recommend as a suitable diagnostic test for DPN.

## Treatment of Diabetic Neuropathy

### Prevention

There is a lack of treatments which reverse the underlying nerve damage causing DPN. Therefore, prevention of DPN is a key component of diabetes care (10). The ADA recommend achieving optimal glucose control in type 1 and type 2 diabetes to prevent or slow the progression of DPN. However, the evidence for enhancing glycemic control in the prevention of DPN is much greater for type 1 than type 2 diabetes (88). Meta-analyses of large, well-conducted randomized controlled trials have identified a clear benefit for optimizing glucose control in type 1 diabetes. For example, the Diabetes Control of Complications Trial (DCCT)/Epidemiology of Diabetes Interventions and Complications (EDIC) study found that intensive therapy significantly reduced the risk of DPN (89). However, the benefits for both glucose and multifactorial risk factor control on DPN are inconclusive in type 2 diabetes (88, 89). Large studies such as the ADDITION-Denmark, UKPDS, Steno-2, and ACCORD trial found intensive glucose and multifactorial treatment had little effect on the incidence of DPN (90–94). However, the presence of multiple comorbidities and risk factors may contribute to the inconsistent findings in these studies (89). Additionally, the types of glucose lowering treatment used may also impact on the results in these studies. Pop-Busui et al. recently found that patients with type 2 diabetes treated with insulin sensitizing therapies had a significantly reduced incidence of DPN compared with insulin providing treatments (10, 95). A meta-analyses of eight randomized studies concluded that there was a trend toward intensive therapy reducing the incidence of DPN in type 2 diabetes, but this did not quite reach statistical significance (*p* = 0.06) (88).

### Pathogenetic Treatments

Pathogenetic treatments of DPN target the underlying disease mechanisms to improve neuronal function. Pathogenetic therapies have shown efficacy in some randomized controlled trials, but the results of pre-clinical studies have largely not translated into clinically meaningful results (96–100). Some of these agents, α-lipoic acid, benfotiamine, actovegin, and epalrestat, are used in some countries (101). However, further robust evidence from clinical trials is necessary before these therapeutic agents can be recommended worldwide (100, 101).

### Symptomatic Treatment of Painful-DPN

**Figure 2:**
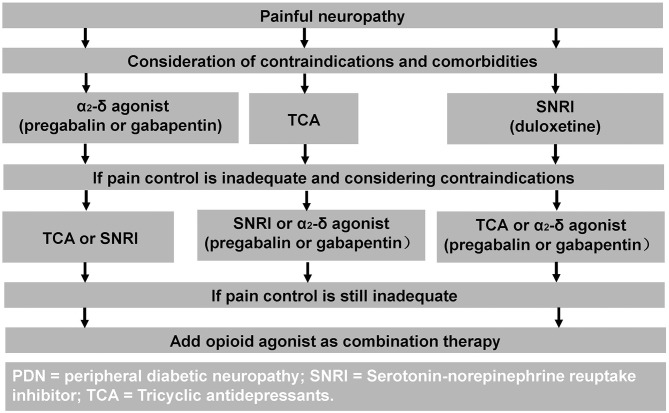
Treatment algorithm for painful-DPN. Reproduced and permission gained from Tesfaye et al. (103).

The mainstay of neuropathic pain treatment in DPN is symptomatic treatment. Unfortunately, pathogenetic treatments and good glycemic control have not been shown to improve neuropathic pain (11). Duloxetine and Pregabalin are the only treatments which have received regulatory FDA approval for the treatment of painful-DPN (10). Whereas, the United Kingdom National Institute of Clinical Excellence recommend Amitriptyline, Duloxetine, Pregabalin, and Gabapentin as first line therapies for neuropathic pain (102). A treatment algorithm is shown in Figure 2 (103).

The α2δ agonists, i.e., gabapentin and pregabalin, are widely recommended, and prescribed agents for painful-DPN. These agents enact their analgesic effect through modulation of the α2δ-1 and α2δ-2 subunits of voltage-sensitive calcium channels (104). Gabapentin is efficacious for the treatment of pain and sleep interference in painful-DPN but has a high rate of side effects, most commonly dizziness, and somnolence (105, 106). The reported number needed to treat to achieve pain relief of at least 50%, is 5.9 (4.6–8.3) (106). Moreover, a network meta-analysis found gabapentin to be the most efficacious and safe therapy for painful-DPN (107).

Pregabalin has linear pharmacokinetics, in contrast to gabapentin, and may be titrated over a short period of time (10, 11). It is the most studied drug for painful-DPN and is recommended as a first line agent by all the major treatment guidelines. It is effective for neuropathic pain and has a side effect profile similar to gabapentin, i.e., dizziness, somnolence, and peripheral oedema (108). In view of the risk of weight gain, and therefore theoretical risk of worsening of metabolic control, Parsons et al. reviewed glycemic/lipid parameters of 11 randomized controlled trials and found no deterioration associated with pregabalin (109). Recent statistics within England and Wales have found an increased number of deaths linked to pregabalin and gabapentin drug misuse prompting a reclassification in the controlling of these medications (110). However, at recommended doses the risk of addiction and dependence for these medications is low in comparison to benzodiazepines, alcohol, and opioids (111, 112). The evidence for other anti-convulsant therapies (e.g., carbamazepine, oxcarbazepine, phenytoin, lamotrigine, and lacosamide) in the treatment of painful-DPN remains limited, but may be effective in some individuals (103).

The other first line pharmacotherapeutic agents for painful-DPN are commonly prescribed anti-depressants, selective serotonin noradrenalin reuptake inhibitors (SNRI) and tricyclic antidepressants (TCA). SNRIs increase the synaptic availability of 5-hydroxytryptamine and noradrenaline increasing the activity of descending pain inhibition pathways (11). Duloxetine is the most widely used agent in this drug class. A Cochrane Collaboration review concluded that at doses of 60 and 120 mg duloxetine is effective in treating painful-DPN, with rare serious side effects (113, 114). The most common side effects include nausea, somnolence, dizziness, constipation, dry mouth, and reduced appetite, although these are commonly mild and transient (104). One of the few comparator drug studies in painful-DPN, the “COMBO-DN” study, found that duloxetine had better efficacy than pregabalin at standard doses (Pregabalin 300 mg/day vs. Duloxetine 60 mg /day) (114).

TCAs have a multimodal analgesic action, including blocking of serotonin and noradrenaline reuptake from synaptic clefts and varying degrees of anticholinergic receptor inhibition (115). Amitriptyline is the most commonly used class of TCA and has been used for neuropathic pain for decades. However, a recent Cochrane Collaboration review and meta-analysis concluded that there is limited evidence for neuropathic pain relief and a poor side effect profile (98, 116). Side effects include dry mouth, constipation, postural hypotension and somnolence, and should be used with caution in elderly patients and those with cardiac disease. Despite its caveats, amitriptyline has been reported to be more effective than placebo in a meta-analysis and remains recommended as a first or second line treatment in all the current guidelines (117).

There are several other treatments which have been studied and are prescribed for painful-DPN with inconclusive evidence. Opioid class medications are an effective means for the treatment of painful-DPN; however, the risk of addiction, side effects and psychosocial complications should limit their use (10). Topical treatments have a theoretical benefit as there is a lower risk of systemic side effects. Agents such as lidocaine patches, capsaicin cream and topical vasodilators however have limited evidence to suggest efficacy (50). For refractory cases of painful-DPN, small studies have found intravenous lidocaine infusions to provide relatively long lasting analgesia (118); however, patients require cardiac monitoring and the treatment is not efficacious in all cases. Open label studies have found vitamin D supplementation to improve neuropathic pain in DPN in patients with vitamin D deficiency (119, 120). Furthermore, non-pharmacological treatments may be considered to complement drug treatments, such as acupuncture, or used as a last resort in resistant cases, such as electrical spinal cord stimulator insertion (103, 121, 122).

### Precision Medicine for Painful-DPN

Unfortunately, the current pharmacotherapeutic agents available for neuropathic pain, including painful-DPN, remain inadequate with the best agents offering only modest improvements in pain which is often offset with significant side effects (13). Traditional neuropathic pain treatments have been prescribed according to disease etiology. However, the clinical features, and perhaps underlying disease mechanisms, of neuropathic pain may vary greatly from individual to individual (123). Recent studies have explored whether stratification according to patient characteristics can identify patients more likely to respond to a particular treatment. The ultimate end goal is “personalized medicine” which is currently only possible in rare cases of neuropathic pain secondary to gene mutations. Over recent years, the stratification methods employed for neuropathic pain treatments include: detailed clinical assessment, sensory profiling, psychology/co-morbidities, physiological changes (e.g., electrophysiology/neuroimaging) and molecular profiling (e.g., genotyping) (14).

### Clinical Phenotype

Somatosensory phenotyping using detailed symptom based questionnaires, such as the Neuropathic Pain Symptom Inquiry (NPSI), or QST may be used to identify patient subgroups reflecting underlying unique nerve mechanistic changes (124). QST is a psychophysical testing method to assess the function of a range of somatosensory modalities. Older techniques such as VPT and thermal thresholds measure large and small fiber function, respectively. However, more recent studies have employed the German Research Network on Neuropathic Pain (DFNS) protocol which quantifies 13 measures of small and large fiber sensory loss and gain abnormalities against normative datasets (125). Using this QST protocol three clusters of somatosensory profiles have been found in neuropathies of varying etiologies (126). Large studies using this QST protocol in DPN have demonstrated sensory loss, particularly thermal hyposensitivity, in painful- compared with painless-DPN (33, 34). However, there is limited data as to whether patient stratification into somatosensory profile clusters predicts response to neuropathic pain treatments. One phenotype-stratified study found that patients with peripheral neuropathic pain and the “irritable nociceptor” phenotype (reserved thermal sensation and gain of sensory function) responded better to oxcarbazepine than those with a non-irritable nociceptor phenotype (127). Moreover, cluster analysis of patient subgroups from the COMBO-DN study using the NPSI found that the addition of pregabalin to duloxetine was effective in patients with pressing and evoked pain, but high dose duloxetine monotherapy was more beneficial for relief of para/dys-aesthesias (128). Additionally, one study showed that conditional pain modulation, a bedside measure of inhibition of experimental pain, predicted duloxetine efficacy in painful-DPN (129). Clinical phenotyping for neuropathic pain, especially using DFNS QST, is receiving enormous attention but further evidence such as positive clinical trials with patient stratification at baseline are required before such phenotyping can be integrated into clinical practice (124).

### Magnetic Resonance Neuroimaging

Central nervous system changes have been well-described in chronic DPN using advanced MR techniques (17). Selvarajah et al. demonstrated that patients with DPN, even those with subclinical DPN, have a lower spinal cord cross-sectional area compared to healthy volunteers and patients with diabetes without peripheral neuropathy (22). Moreover, DPN is associated with peripheral brain gray matter volume loss localized to the primary somatosensory cortex, supramarginal gyrus, and cingulate cortex (23). Quantification of cerebral metabolites using proton MR spectroscopy (1H-MRS) has demonstrated reduced N-acetyl aspartate:creatine ratio suggesting neuronal dysfunction within the thalamus in DPN (130). Additionally, an imbalance of the cerebral neurotransmitters glutamate/glutamine and gamma-aminobutyric acid has been found in the posterior insula in DPN (131).

Neuroimaging has identified a number of neurochemical, structural, neurovascular, and functional alterations secondary to chronic pain diseases. In painful-DPN, studies have shown increased thalamic microvascularity (132), impaired spinal inhibitory function (133), and altered functional connectivity between brain regions involved in pain processing (134). Moreover, MR alterations are related to different clinical phenotypes in painful-DPN (135). A recent study found that patients with insensate painful-DPN, compared to groups with painless-DPN and sensate painful-DPN, had lower somatosensory cortical thickness and expansion of the homuncular area representing pain. Limited studies have also demonstrated that cerebral alterations may be predictive of response to pain treatments (136). Watanabe et al. assessed the cerebral blood flow of patients with and without painful-DPN and longitudinally assessed flow changes after treatment with duloxetine (137). They found that greater baseline cerebral blood flow within the anterior cingulate cortex was associated with better pain relief. However, a recent study found that neurometabolites measured using 1H-MRS in painful-DPN were not significantly altered between placebo and pregabalin, but small differences were observed between pregabalin doses (138). Although, cerebral alterations have been described in painful-DPN, further study of biomarkers as clinical endpoints is required (17, 49).

### Genetic

The increased efficiency and availability in gene sequencing technology has led to the exploration of potential genetic factors predisposing to a number of chronic diseases. A meta-analysis found that variants in several genes, e.g., HLA, COMT, OPRM1, TNFA, IL-6, and GCH1, were associated to neuropathic pain in at least one study (139). Moreover, genetic variants have been associated with DPN and neuropathic pain in diabetes (12). Genome-wide association studies have found a number of gene polymorphisms related to painful-DPN (140, 141). Moreover, rare voltage-gated sodium channel Nav 1.7 genetic variants have been shown to be associated with small fiber neuropathy and painful-DPN (142, 143). With the development of the voltage-gated sodium channel Nav 1.7 research, more mutation sites related to painful-DPN have been found, but the mutation perhaps needs further validation (144). Interestingly, Blesneac et al. found 10 out of 111 patients with painful-DPN harbored rare Nav 1.7 variants and these patients reported more severe pain and increased sensitivity to pressure stimuli on QST (145). These findings indicate a link between clinical phenotype and genetic variants which may predict response to treatment. Furthermore, a recent study found that patients with Nav 1.7 mutations and small fiber neuropathy treated with the anticonvulsant lacosamide had significantly improved pain compared with placebo (146).

## Conclusions

The prevalence of diabetes mellitus is rising to epidemic proportions. Consequently, there will be a dramatic rise in the numbers of patients suffering with its chronic complications, including DPN. The current management strategy for DPN is focused upon early detection of the condition and prevention of diabetic foot syndromes. New diagnostic techniques may aid the clinical assessment in detecting clinical and subclinical DPN, but further research is required to determine whether clinical outcomes such as foot ulceration, amputation and cardiovascular disease can be prevented with their routine use and whether they may be used as surrogate end points for DPN.

Up to half of patients with DPN suffer with neuropathic pain. Our understanding of why some patients develop painful neuropathic symptoms is unclear. Our current treatments aim to alleviated symptoms, but at best reduce pain scores by 30–50% in about a third of cases. However, the failure of drug trials may be as a result of the empirical use of treatments whereas a more individualized approach by using patient characteristics (e.g., clinical phenotypes, cerebral biomarkers or genotype, etc.) to stratify patients may be more effective (14). However, further validation is required before any of these factors can be considered for stratification in clinical practice but there is potential that it may improve patient outcomes in painful-DPN.

## Author Contributions

HY: prepared and wrote up the draft. GS: prepared and majorly revised the draft. YY: prepared the paragraphs on DPN precision medicine. SW: prepared the paragraphs on DPN treatment. BD: prepared the reference list. ST: revised the draft. LG: prepared, wrote up, and revised the manuscript.

## Conflict of Interest

The authors declare that the research was conducted in the absence of any commercial or financial relationships that could be construed as a potential conflict of interest.
